# Association between the body roundness index and female infertility: a cross-sectional study from NHANES

**DOI:** 10.3389/fendo.2025.1504878

**Published:** 2025-01-31

**Authors:** Ming Liu, Yifang Zhang, Jian Liu

**Affiliations:** Department of Gynecology and Oncology, First Affiliated Hospital, Bengbu Medical University, Bengbu, Anhui, China

**Keywords:** NHANES, infertility, body roundness index (BRI), obesity, cross-sectional studies

## Abstract

**Background:**

Infertility is strongly associated with obesity. The body roundness index (BRI) is a more accurate assessment of visceral fat content than the body mass index (BMI). However, current evidence on the association between visceral fat accumulation and infertility remains insufficient and controversial. Therefore, we utilized the 2017-2020 National Health and Nutrition Examination Survey (NHANES) database to explore the correlation between BRI and infertility.

**Methods:**

We used multiple logistic regression, smoothed curve fitting, subgroup analyses, and interaction tests to investigate the potential association between BRI and infertility. Additionally, we assessed the ability of BRI and BMI to predict infertility risk using receiver operating curve (ROC) analysis and calculate the area under the curve (AUC),sensitivity, and specificity.

**Results:**

In the study, 1463 women aged 20 to 45 participated, and 172 of them were found to be infertile. After adjusting for all factors except body measurements, the findings indicated that for each one-unit increase in BRI, there was a 19% increase in the risk of infertility (OR = 1.19, 95% CI 1.05, 1.34). The analysis also revealed a positive nonlinear relationship between BRI and infertility. Furthermore, based on the ROC curves, it was observed that BRI was a more reliable predictor of infertility risk compared to BMI (BRI AUC = 0.5773, BMI AUC = 0.5681).

**Conclusion:**

This study demonstrated a positive association between higher BRI values and infertility among adult women in the United States and showed a stronger association than BMI.

## Introduction

Infertility is a medical condition that prevents pregnancy after more than one year of regular sexual intercourse without any contraception (1). According to the World Health Organization report, infertility affects about 17.5% (2) of the population of reproductive age, causing a substantial psychological and financial burden on the patients (3–5).

With lifestyle changes, obesity has become one of the critical risk factors for infertility (6). Although widely used, traditional measurements such as BMI have gradually revealed their limitations in reflecting body fat distribution (7). In 2013, Thomas DM et al. (8) proposed the BRI as a new predictor of visceral adipose tissue and percentage of body fat, which estimates total body fat and body mass by considering the human body as an ellipsoid and combining height and waist circumference to calculate the percentage of total and localized fat, responding to the individual’s body shape characteristics.

This study aims to investigate the association between BRI and infertility and whether it is superior to BMI. It will help healthcare professionals to better identify and manage women at high risk of infertility and provide a basis for future research on the relationship between visceral fat accumulation and women’s reproductive health.

## Materials and methods

### Survey description

The National Health and Nutrition Examination Survey (NHANES) is a comprehensive national survey conducted by the Centers for Disease Control and Prevention (CDC) to evaluate the health and nutritional status of the U.S. population. Due to its use of probability sampling, NHANES data is considered highly credible and valuable for academic research. The 2017-2020 NHANES serves as the primary data source for this analysis. The National Center for Health Statistics (NCHS) Ethics Review Board has approved all study protocols for NHANES, and each participant has provided written informed consent. Detailed NHANES study designs and data are publicly available at http://www.cdc.gov/nchs/nhanes/.

### Study population

**Figure 1:**
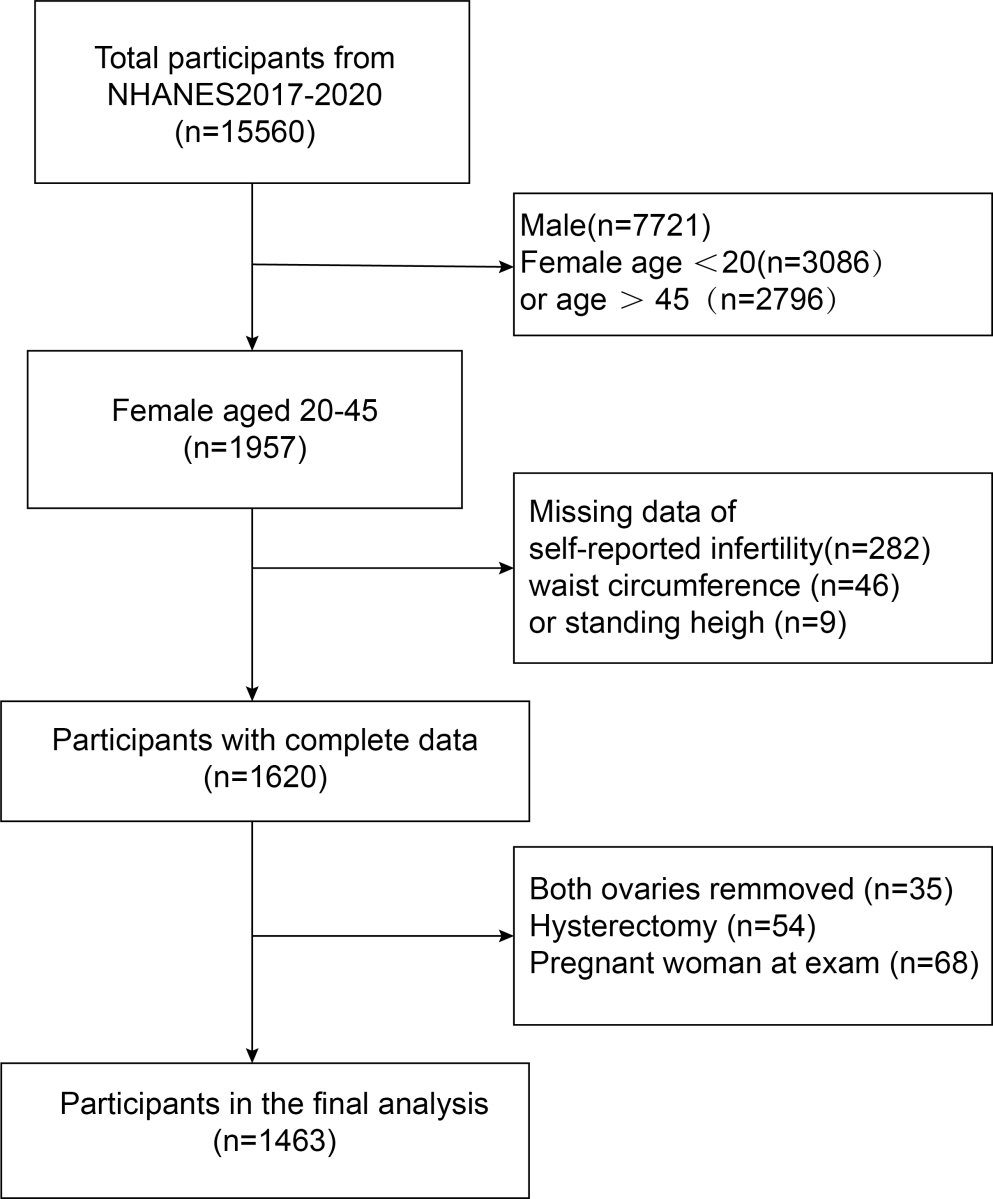
Flow chart for participants recruitment, NHANES 2017-2020.

Initially, 15,560 participants from the 2017-2020 NHANES were enrolled. In order to select the most eligible subjects for the current study, the researchers further evaluated these individuals. First, they excluded 7,839 male subjects and female subjects younger than 20 years (n = 3,086) or older than 45 years (n = 2,796). Second, participants with missing data on self-reported infertility (n = 282) and body mass index (n = 55) were excluded. Participants who had both ovaries removed (n = 35), underwent hysterectomy (n = 54), or were pregnant at the time of examination (n = 68) were also excluded. Ultimately, 1,463 eligible women participated in the study (**Figure 1**).

### Exposure and ending definition

The exposure factor was BRI, which was calculated using the formula: $BRI=364.2-365.5\times \sqrt{1-\left\{\frac{{\left[WC(m)/2\pi \right]}^{2}}{{\left[0.5\times Height(m)\right]}^{2}}\right\}}$ (8, 9). The height and waist circumference data were obtained from the participants’ physical examination documents. Body measurements were taken by professionally trained health technicians at the Mobile Health Screening Center and were recorded with the corresponding person to ensure accuracy. Participants were asked to remove their clothes and shoes before the measurements were taken. Height was measured while standing, and waist circumference was measured at the midpoint between the lower ribs and the upper part of the hips in a standing position.

The outcome indicator is self-reported female infertility, which was obtained from the Reproductive Health Questionnaire. The questionnaire was administered by professionally trained interviewers using the Ministry of Education’s computer-assisted personal interview system. Participants were asked about their pregnancy history with the question, “Have you been trying to get pregnant for one year?” Women who answered “yes” were categorized as having infertility, and those who answered “no” were categorized as not having infertility (Questionnaire variable name: RHQ074).

### Covariates

Covariates in this study included age, race, education level, marital status, poverty-to-income ratio (PIR), smoking status, hypertension, diabetes mellitus, regular menstruation, sex hormone use, history of treatment for pelvic infection, waist circumference, and BMI. In our analyses, we used the questionnaire “Your doctor told you that you have diabetes” to determine if participants had diabetes. Those who answered “yes” were categorized as diabetic. Similar questionnaires were used to identify patients with hypertension and other conditions. Additionally, we used the questionnaire “Have you smoked at least 100 cigarettes in your life” to determine smoking status, categorizing those who answered “yes” as smokers. The above covariates were determined professionally after reviewing existing studies. All data collection and measurement procedures for these covariates are available on the NCHC website. Moreover, measurement procedures for these covariates are available on the NCHC website.

Questions about missing data, after data processing, the only variable with missing values was the poverty-to-income ratio (PIR), with a missing data percentage of 10.2% (168/1463). The missing values were estimated using the continuous iterative imputation method, which is based on the conditional distribution of other variables. This approach improves the accuracy and reliability of subsequent analysis.

### Statistical analysis

Statistical analyses were performed using Empower Stats software (X&Y et al., USA) and the R package (version 3.4.3). p < 0.05 was considered statistically significant. Data were obtained from NHANES 2017-2020. The NHANES study employed a sophisticated multi-stage probability sampling design to collect data, which was taken into account in our analysis. To ensure representativeness of the sample, we performed weighted multivariate logistic regression analyses. Demographic and measurement indicators were descriptively analyzed for the study population. These indicators were categorized into two groups according to the presence or absence of infertility. Continuous variables were expressed as Mean ± Standard Error (Mean ± S.E.), and a t-test was used to compare groups. Categorical variables were expressed as frequencies (constitutive ratios) [n (%)], and comparisons between groups were made using the chi-square test. Multivariate regression models were used to examine the association between BRI and infertility. In model 1, the covariates were not adjusted. In model 2, adjustments were made for age and race. Model 3 was adjusted for the following factors: age, race, marital status, education level, household income, poverty rate, smoking at least 100 cigarettes, diabetes, hypertension, previous use of female hormones, previous treatment for pelvic infection, and regularity of menstruation in the past 12 months. Model 3 also used the RCS curve to assess the association between BRI and infertility. In addition, subgroup analyses and interaction tests were performed according to age, marital status, smoking status, diabetes, and regularity of menstruation. We aim to identify possible impact modifiers and understand the differences in results among various groups and circumstances. The sensitivity of BRI and BMI in predicting infertility was assessed by examining the receiver operating characteristic curve (ROC) and calculating the area under the curve (AUC).

## Results

### Baseline characteristics of participants

In **Table 1**, we can see the characteristics of participants aged 20 to 45 years based on whether they had infertility. Out of 1463 participants, 172 (11.76%) were found to be infertile. The research showed that older women who lived with a partner who smoked at least 100 cigarettes, had diabetes mellitus, irregular menstruation, higher waist circumference, body mass index, and body roundness index reported a higher prevalence of infertility.

**Table 1:** Weighted demographic characteristics of selected participants from the NHANES 2017-2020.

Variables	Non-infertility	Infertility	*P*-value
Age (years)	31.74 ± 7.51	33.17 ± 6.45	0.0208
Race/ethnicity(%)			0.4653
Mexican American	11.85	12.02	
Other Hispanic	8.19	12.12	
Non-Hispanic White	55.24	54.58	
Non-Hispanic Black	13.95	13.10	
Other Race	10.77	8.17	
Education level (%)			0.1608
Less than 9th grade	2.56	2.00	
9-11th grade	5.83	9.47	
High school graduate/GED or equivalent	22.34	25.10	
Some college or AA degree	30.83	33.24	
College graduate or above	38.43	30.19	
Marital status(%)			<0.0001
Living with Partner	56.23	78.17	
Live alone	43.77	21.83	
Ratio of family income to poverty	2.82 ± 1.68	2.79 ± 1.57	0.8401
Smoked≥100 cigarettes in life(%)			0.0003
Yes	30.22	44.24	
No	69.78	55.76	
Hypertension(%)			0.8819
Yes	10.87	10.63	
No	88.98	89.37	
Not recorded	0.15		
Diabetes(%)			<0.0001
Yes	1.90	9.49	
No	96.77	88.73	
Borderline	1.16	1.78	
Not recorded	0.17		
Regular periods(%)			0.0249
Yes	92.81	87.80	
No	7.19	12.20	
Ever use female hormones(%)			
Yes	4.68	4.03	0.8453
No	95.20	95.97	
Not recorded	0.12		
Ever treated for a pelvic infection(%)			0.2143
Yes	4.24	7.13	
No	95.54	92.87	
Not recorded	0.22		
Waist Circumference (cm)	94.47 ± 18.97	102.99 ± 20.49	<0.0001
Body Mass Index (kg/m2)	29.32 ± 8.63	32.52 ± 9.40	<0.0001
Body Roundness Index/BRI	5.41 ± 1.40	5.89 ± 1.43	<0.0001

Data in the table: For continuous variables: survey-weighted mean (95% confidence interval), P-value was by survey-weighted linear regression (wtmecprp). For categorical variables: survey-weighted percentage (95% confidence interval), P-value was by survey-weighted Chi-square test (wtmecprp).

### Associations between BRI and risk of infertile

**Figure 2:**
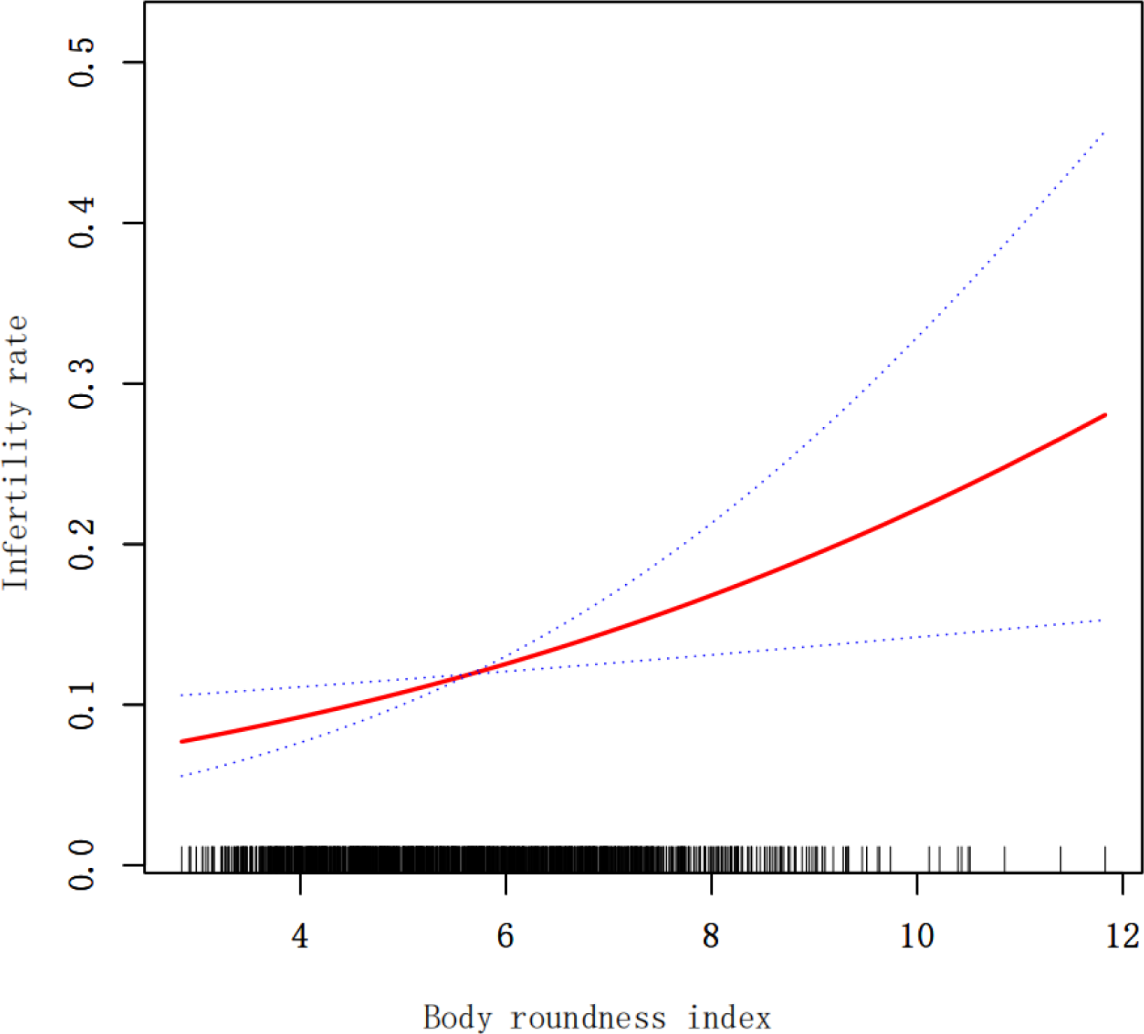
Association between BRI and infertility rate.

In **Table 2**, it is evident that there is a positive association between BRI and the prevalence of infertility. This positive correlation remained consistent in model 3 (OR 1.19; 95% CI 1.05-1.34), indicating that each unit increase in BRI raises the risk of infertility by 19%. For sensitivity analysis, we converted BRI from a continuous variable to a categorical variable (quartile). The likelihood of infertility in Q4 was 83% higher compared to Q1. However, the difference between Q2 and Q1 was not statistically significant (OR 1.17; 95% CI 0.68-2.02), nor was the difference between Q3 and Q1 (OR 1.52; 95% CI 0.89-2.59). Additionally, we used smoothed curve fitting based on model 3 to investigate the relationship between BRI and infertility, revealing a positive nonlinear relationship (**Figure 2**).

**Table 2:** Association between the body roundness index and female infertility.

Exposure	OR (95%CI), *P*-value	Model 2	Model 3
	Model 1		
BRI	1.19 (1.07, 1.33) 0.0010	1.18 (1.06, 1.32) 0.0036	1.19 (1.05, 1.34) 0.0070
BRI Quartile			
Q1, [2.845, 4.534]	1(Reference)	1(Reference)	1(Reference)
Q2, [4.539, 5.474]	1.33 (0.81, 2.18) 0.2571	1.24 (0.75, 2.05) 0.3971	1.17 (0.68, 2.02) 0.5621
Q3, [5.476, 6.581]	1.48 (0.91, 2.40) 0.1148	1.38 (0.84, 2.27) 0.1989	1.52 (0.89, 2.59) 0.1276
Q4, [6.585, 11.827]	1.99 (1.25, 3.17) 0.0036	1.85 (1.14, 2.99) 0.0122	1.83 (1.08, 3.11) 0.0254

Model 1: no covariates were adjusted;

Model 2: age and race were adjusted;

Model 3: age, race, education level, marital status, ratio of family income to poverty, smoked≥100 cigarettes in life, hypertension, diabetes, regular periods, ever use female hormones, ever treated for a pelvic infection were adjusted;

BRI, body roundness index; OR, odds ratio; CI, confidence interval.

### Subgroup analyses

**Figure 3:**
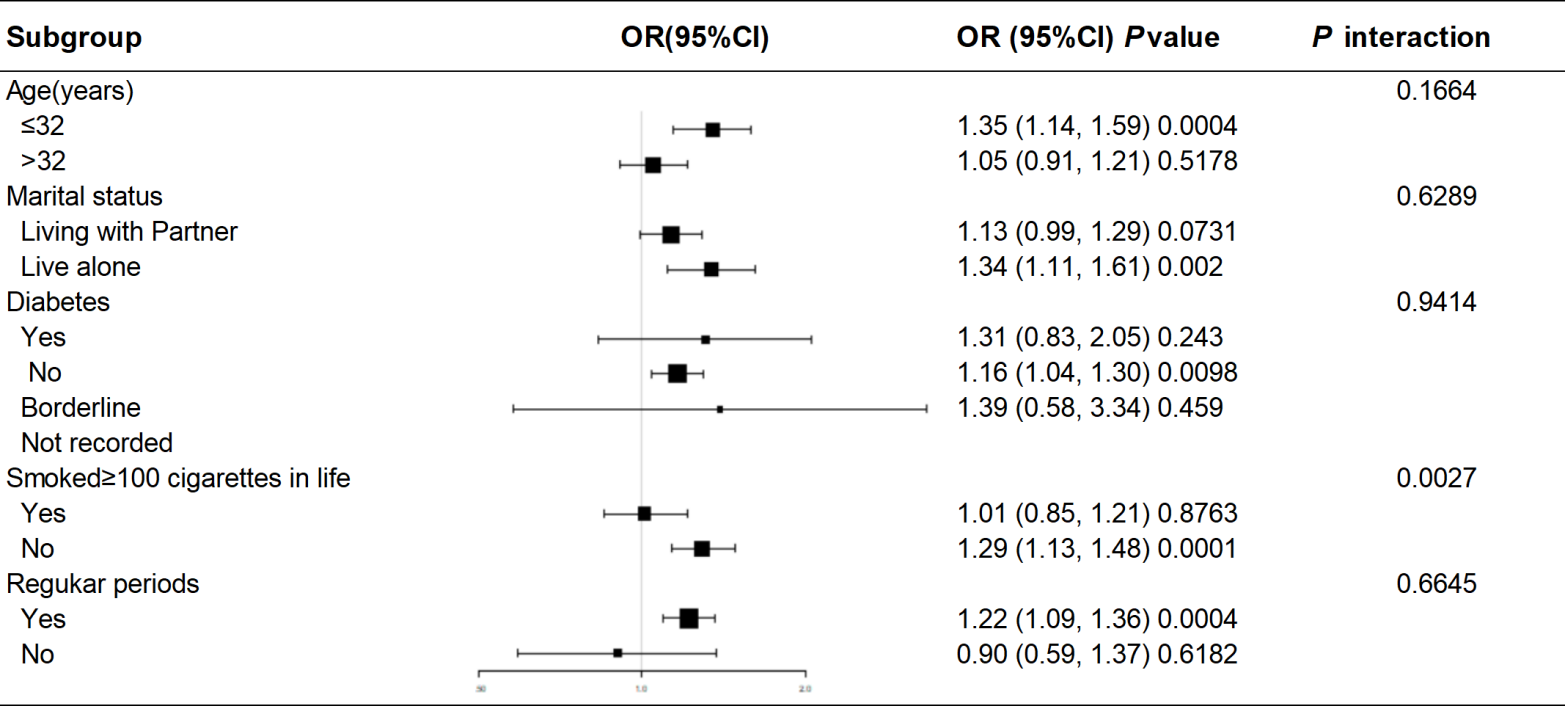
Subgroup analysis for the association between BRI and female infertility.

Subgroup analyses and interaction tests were performed using different variables to investigate the stability of the association between BRI and female infertility across subgroups. Subgroup analyses were stratified by age, marital status, diabetes, smoking status, and menstrual pattern. In all subgroups, BRI values were generally and consistently positively associated with the risk of infertility. The smoking group showed a significant interaction effect (P for interaction=0.0027), suggesting that smoking or not smoking may have a differential effect on the relationship between BRI and infertility (**Figure 3**).

### Comparison of BRI and BMI in predicting infertility

**Figure 4:**
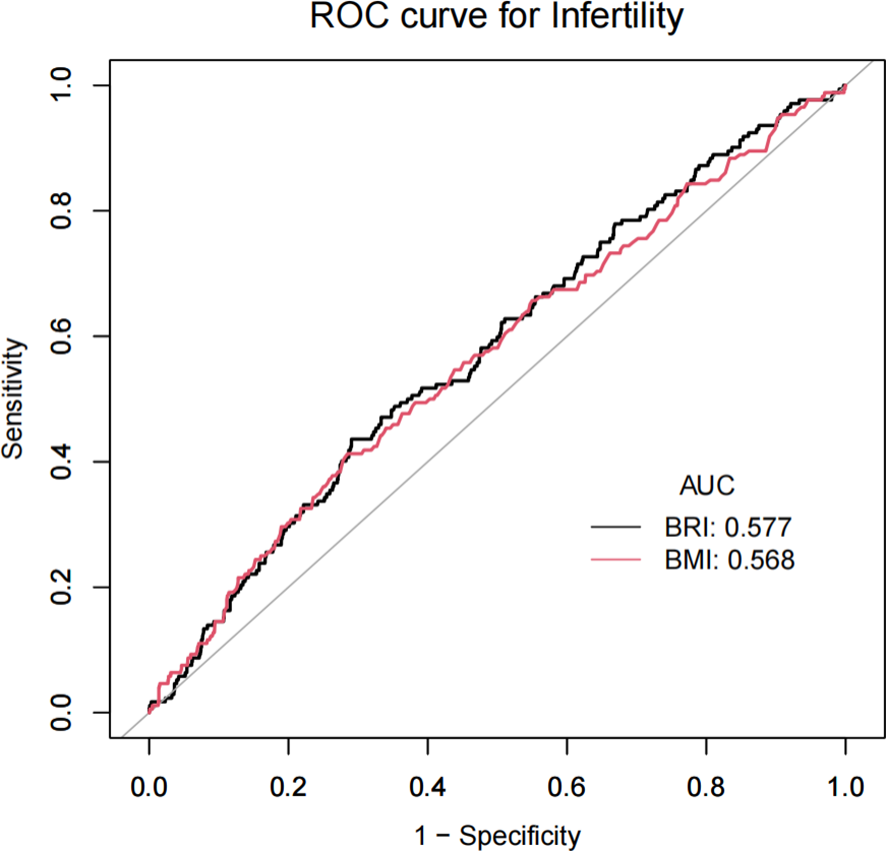
Comparison of ROC curves for BRI and BMI in predicting infertility.

In this study, we initially calculated the sensitivity and specificity at varying thresholds, subsequently employing the Youden index to assess the performance of BRI and BMI at distinct thresholds. Youden’s index = sensitivity + specificity - 1.We identified the optimal cut-off point through the selection of a threshold that maximized the Youden index. This process ensured an optimal balance between positive and negative predictions. The results demonstrate that BRI exhibits a Youden index of 14.48%, an optimal threshold of 6.2945, an AUC of 0.5773, a sensitivity of 43.60%, and a specificity of 70.95%. The Youden index for BMI is 12.77%, with an optimal threshold of 3.345 and an AUC of 0.5681. The sensitivity is 41.28%, while the specificity is 71.49%. BRI demonstrated superior performance relative to BMI. Furthermore, a receiver operating characteristic (ROC) curve was plotted. The receiver operating characteristic (ROC) curve illustrates the correlation between sensitivity and the false positive rate (1-specificity) at varying thresholds. The optimal inflection point is defined as the point on the receiver operating characteristic (ROC) curve that is furthest from the diagonal line (**Table 3**, **Figure 4**).

**Table 3:** Comparison of ROC curves for BRI and BMI in predicting infertility.

Anthropometric Measures	Youden index	Best thresholds	Sensitivity	Specificity	AUC(95%CI)	*P*-value
BRI	0.1448	6.2945	0.4360	0.7095	0.5773 (0.5321,0.6225)	0.0010
BMI	0.1277	33.4500	0.4128	0.7149	0.5681 (0.5215,0.6147)	0.0024

## Discussion

In this cross-sectional study, which included 1,463 women of childbearing age attending NHANES in the United States from 2017-2020, of whom 172 reported infertility (prevalence 11.76%), we found a positive nonlinear association between BRI and infertility. After adjusting for all covariates except body measurements and converting the BRI to categorical variables by quartiles (Q1-Q4), the positive association remained, with a 19% increase in infertility risk for each unit increase in the BRI. Individuals with a high BRI score (Q4) had a higher probability of developing infertility compared to Q1. In subgroup analyses and interaction tests for age, marital status, diabetes mellitus, smoking status, and menstrual regularity, the smoking group showed a significant interaction effect (P for interaction=0.0027), suggesting that smoking status may modulate the effect of BRI on infertility. To further explore the ability of the BRI to predict infertility, we performed a ROC analysis and compared the BRI with BMI in predicting infertility. The BRI was found to be superior to BMI in its ability to predict infertility.

In this study, BRI was used as a potential predictor of female infertility in the NHANES cohort. Although previous studies have identified a link between obesity and reproductive dysfunction (10, 11), our study highlights that BRI is more effective than BMI in capturing the effects of central obesity. This distinction is critical because central obesity is more closely associated with metabolic disorders (12), which can impair reproductive function (13). Prior research has indicated that smoking may potentially induce apoptosis of ovarian follicle cells in women, which could subsequently affect the anatomy and function of the uterus and fallopian tubes, among other reproductive organs. This may consequently lead to an increased risk of infertility (14–16). He et al. analyzed data from 3,665 female participants aged 18–45 in the NHANES (2013-2018) study and found that the risk of infertility in smokers was 41.8% higher than in never smokers. However, the results of the subgroup analysis demonstrated that the relationship between smoking status and infertility varied across different ethnic groups and age categories. In particular, the study revealed that the correlation between smoking and infertility was only significant for women aged 25–38 and Mexican Americans (17). The findings of our research demonstrate that BRI exerts a more pronounced effect on the likelihood of infertility in women who do not engage in smoking. The observed correlation between smoking and infertility suggests a possible mechanism by which the two may be associated, but the exact nature of this mechanism requires further investigation. The identification of interactions between smoking and BRI provides new insights into how lifestyle factors influence the relationship between body composition and fertility. Research discussing the positive association between higher BRI and increased risk of infertility is consistent with existing literature on the effects of obesity on reproductive health. Excess adipose tissue disrupts hormonal balance (18–20), particularly affecting the hypothalamic-pituitary-ovarian axis, which is critical for ovulation (21). The superior diagnostic efficacy of BRI compared to body mass index (BMI) highlights its potential role in the clinical setting. BMI does not differentiate between muscle and fat mass (22), nor does it consider fat distribution. In contrast, BRI provides a more nuanced assessment (23) that better identifies women at risk of infertility due to metabolic complications.As an emerging health indicator, BRI has broad applicability in various contexts. A large retrospective study has demonstrated that an elevated BRI represents a significant risk factor for cardiovascular disease, and the correlation between the two remains statistically significant even after adjusting for potential confounding factors (24). Additionally, inadequate cardiovascular health may result in insufficient blood and nutrient supply to the ovaries and uterus, potentially affecting fertility (25). This provides further evidence for the existence of complex and interrelated pathophysiological mechanisms linking obesity, cardiovascular health, and reproductive function, which are worthy of further research.

The present study should be recognized as having certain limitations. First, its cross-sectional nature limits causal inferences; a longitudinal study is necessary to determine the temporal relationship between BRI and infertility. Secondly, it should be noted that, although the present study focused on the effect of BRI on infertility, it is acknowledged that the occurrence of infertility is a complex phenomenon involving a multitude of factors in both men and women. Consequently, the failure to consider both factors in their entirety represents a limitation of this study. In addition, reliance on self-reported factor measures may have introduced information bias in our findings. Although we adjusted for some confounders, residual confounders from unmeasured variables such as diet or physical activity could not be excluded.

## Conclusion

The results of the study indicate that an elevated BRI is associated with an increased risk of infertility. This association is stronger than that observed between BMI and the risk of infertility. BRI, as a novel indicator of obesity, may serve as a valuable reference indicator for individuals at elevated risk of infertility.

## Data Availability

The datasets presented in this study can be found in online repositories. The names of the repository/repositories and accession number(s) can be found in the article/supplementary material.
